# Phthisis Pulmonalis

**Published:** 1855-10

**Authors:** Henry Fisher

**Affiliations:** 52 Amity Street, N. Y.


Phthisis Pulmonalis.—The following article we transfer to our pages from the *New York Medical Times:*

*Gentlemen.*—In reference to the employment of *Iodide of Ethyle* in the treatment of Phthisis Pulmonalis in your March number, I may state in continuation of the subject, the leading features of four well-marked cases of phthisis in which I have employed it. I have also given it in several other cases: in one, it had to be abandoned owing to the production of anæsthesia by very small doses; in some, the phthisical symptoms were not marked with sufficient distinctness to afford evidence of its value; and in others, it was used so irregularly, or for such a short time, that no definite conclusion could be drawn regarding its effects; and I may mention, in passing, that two circumstances militate very strongly against the regularity of its employment by patients; some concluding from the smallness of the dose, and its simple mode of administration, that its influence could be but trifling, while others are deterred from its use by a suspicion of the whole class of inhaling remedies—an amount of caution which is unfortunately too well grounded, owing to the manner in which this mode of treatment has been paraded before the public. The first case I shall mention is that of H. P., a young man aged 19, who commenced the use of the ethyle on the first of December, 1854, at which time he presented the following symptoms: Had suffered from cough for eight months, the cough being attended latterly with muco-purulent expectoration; the sputa were frequently streaked with blood, and once about half an ounce of what was described as being unmixed arterial blood, was raised; there was some dyspnœa, and considerab.e pleurodynic pain after exercise; nocturnal hectic was of tolerably frequent occurrence; and there was general lassitude and debility. There was marked dullness on percussion, anteriorly, on the right side of the thorax, extending from the second to the fourth intercostal spaces, where also there was obscuration or obliteration of the natural, respiratory murmur; moist, mucous râle and ronchus were audible; and there was pretty distinct broncophony. In other portions of the right side, and over the left side of the thorax, the respiration was exaggerated and puerile, but otherwise presented no indications of disease. He had taken cod-liver oil, and employed counter irritation over the affected lung, without much amelioration of the thoracic symptoms, but he had gained somewhat in weight. He now commenced inhaling the iodide of ethyle, using fifteen minims three times a day, and continued it regularly till the 20th of Feb., 1855, when it became necessary to suspend its use, owing to diarrhœa, frontal headache, nausea, and other symptoms of iodism having made their appearance. Previous to these symptoms, and after their subsidence, which took place shortly on the suspension of the remedy, his condition was and has been most satisfactory; he has scarcely ever coughed, there has been no return of hectic or hæmoptysis, and he has been gaining flesh and strength rapidly. The chest, on percussion over the affected portion, exhibits but very slight dullness, and the respiratory murmur approaches very nearly a natural character.

In another case which presented the symptoms of tubercle in its earliest stage, accompanied with very severe bronchitis, the use of the ethyle for a few days, combined with counter-irritation, confinement, and a strict antiphlogistic regimen, sufficed to effect the complete suppression of the cough and dyspnœa, which, as yet, after an interval of some months, have not reappeared.

The other two cases in which I have had an opportunity of testing its utility with regularity, presented unmistakable evidence of the existence of a small cavity immediately under the clavicle, in the one case occupying the right, in the other the left lung. In both of these cases the severity of the symptoms has been much mitigated, and the progress of the disease arrested; and I believe it to be possible that a cicatrizing process may yet be established.

It will be urged that equally good results in cases of phthisis have been secured by the use of counter-irritation, the administration of cod-liver oil, and strict attention to the hygienic and dietetic rules which form what may be called the routine treatment of the disease; and this I think no one will be inclined to deny; but, at the same time, I am convinced that the iodide of ethyle will be found, in most cases, a most valuable adjuvant, and that the practical results will be found fully to bear out the theoretical conclusion drawn from a consideration of its chemical composition. Its action may take place in two ways: it must, in the first instance, exert a direct sedative local action on the diseased part; and secondly, from absorption, a general or constitutional action; and it is not unreasonable to suppose that it exerts an influence in the early stages of tubercular diseases by promoting absorption, and later, by aiding cicatrization.

I do not doubt that the real and primary cause of phthisis is still to be sought for in defective action of the organs of assimilation; and also that its ultimate cure, if ever, is to be found in the restoration of the healthy discharge of these functions; and that it is to the attainment of this object that our efforts should be mainly directed. This must of necessity be a work of time; and I believe that the iodide of ethyle while aiding the measures which we employ for this purpose by its general alterative and tonic action, is also, by its local action on the diseased part, gaining us the time necessary for the full development of our plans.

The expensive nature of the substance, and the difficulty of procuring it in sufficient purity, form at present a serious barrier to its employment, and have induced some, I believe, to use in its stead a mechanical solution of iodine in sulphuric ether. If the employment of this mixture is attended with any benefit it must be owing entirely to the sedative action of the ether, as very little if any of the iodine will become volatilized; a fact of which any one may easily convince himself by placing a portion of the solution in a watch glass, evaporating dish, or other vessel, previously weighed, permitting evaporation to take place, when the sides of the vessel will be found coated with iodine and it will have gained in weight in exact proportion to the quantity previously held in solution by the ether. I do not know of any indications which forbid a trial of the iodide of ethyle unless it be in those rare cases where very small doses produce anæsthesia. It has been given with great advantage in many cases of chronic bronchitis, and also by the mouth in some cases of rheumatism and other diseases where iodine is an appropriate remedy. It is not improbable that others of the compounds of the organic radicles are endowed with equally valuable medicinal properties; and the subject presents a wide and interesting field for therapeutical investigation.

52 *Amity Street, N. Y.* HENRY FISHER, M. D.

